# Knockdown of Aurora kinase B alleviates high glucose-triggered trophoblast cells damage and inflammation during gestational diabetes

**DOI:** 10.1515/biol-2022-1031

**Published:** 2025-03-06

**Authors:** Yuzhuo Ma, Yongyun Shi, Yujie Liu

**Affiliations:** Department of Gynecology and Obstetrics, The First Affiliated Hospital of University of Science and Technology of China (Anhui Provincial Hospital), No. 17 Lujiang Road, Luyang District, Hefei, Anhui, 230001, China

**Keywords:** gestational diabetes, trophoblast, AURKB, inflammation, PI3K/Akt

## Abstract

This research investigates how Aurora kinase B (AURKB) functions in trophoblast cells when they are exposed to high levels of glucose during gestational diabetes. The findings from RT-qPCR and western blotting show that when in a high-glucose environment, AURKB expression increases in both the placenta and trophoblast cells of patients with gestational diabetes mellitus. Additionally, when AURKB is silenced in high-glucose conditions, it leads to boosted proliferation of trophoblast cells and reduced inflammation. Knockdown of AURKB inhibits the expression of phosphoinositide 3-kinase (PI3K)/protein kinase B (AKT) pathway in high glucose (HG) environment. Knockdown of AURKB may ameliorate injury and inflammatory responses in HG-exposed trophoblast cell lines in part by regulating the PI3K/AKT signaling pathway.

## Graphic abstract

**Figure:**
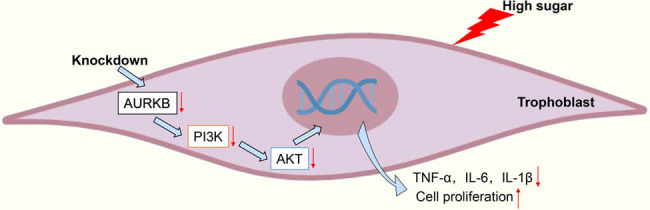

## 1 Introduction

In recent years, there has been a rise in the prevalence of gestational diabetes mellitus (GDM), a condition characterized by disrupted glucose metabolism and increased insulin resistance during pregnancy. It now impacts around 5–20% of pregnancies, demonstrating a growing trend [1,2]. Pregnancy-related body mass index in the overweight or obese range, advanced mother age, smoking, family history of diabetes, and any form of diabetes are proven risk factors for GDM [3]. Female individuals diagnosed with GDM are at a higher risk of experiencing chronic conditions like type 2 diabetes, high blood pressure, vascular issues, and nonalcoholic fatty liver disease. Moreover, children born to these individuals face an elevated likelihood of developing type 2 diabetes or obesity [4,5]. Given the possible negative repercussions for both mother and child, it is critical to investigate the pathophysiology of GDM.

In pregnancies complicated by GDM, the placenta exhibits trophoblastic dysfunction in contrast to pregnancies without complications [6]. High blood sugar levels during GDM not only impact the baby, but also the growth and performance of the placenta. The specific reasons behind GDM are still unclear, but some research indicates that abnormalities in the way trophoblast cells differentiate, invade, multiply, die, and regulate their growth cycle may play a role. These functions are essential for the proper formation of the placenta [7,8]. Examining the trophoblast dysfunction caused by high glucose (HG) could be a useful approach in researching the etiology of GDM.

Several research studies have highlighted the association between Aurora kinase B (AURKB) and diabetic nephropathy [9]. Additionally, research has indicated that AURKB is associated with compromised early pregnancy progression [10]. Based on findings from gene co-expression network analysis, AURKB has the potential to serve as a valuable biomarker for gestational diabetes [11]. AURKB, a key serine/threonine kinase, plays a vital role in regulating cell division and the distribution of chromosomes [12–14]. Its levels are increased in various cancer types such as lung, breast, pancreatic, ovarian, and prostate cancers. Targeting AURKB is increasingly recognized as a hopeful strategy for treating multiple cancer types [13]. However, the role and mechanism of AURKB in gestational diabetes are rarely reported.

Research has shown a close relationship between the development and advancement of GDM with the phosphoinositide 3-kinase (PI3K)/protein kinase B (AKT) signaling pathway. This pathway, a crucial insulin signaling pathway, has been extensively studied in the investigation of diabetes pathogenesis [15]. Studies have shown that AURKB functions through the PI3K/AKT pathway. For example, AURKB can promote cancer proliferation by inducing epithelial-mesenchymal transition through the PI3K/AKT signaling cascade [16].

In order to replicate GDM conditions *in vitro*, we employed HG (30 mM glucose)-induced trophoblast cells (HTR-8/SVneo cells) in this investigation. Our objectives were to look into the precise function of AURKB in GDM and to research any possible pathways that AURKB may have in the malfunctioning of placental trophoblasts in GDM.

## 2 Method

### 2.1 Human plasma collection

All placental samples were taken at the First Affiliated Hospital of University of Science and Technology of China (Anhui Provincial Hospital). Placenta samples were collected from ten GDM patients and ten healthy individuals. Placental samples were treated with a TRIzol kit (Invitrogen, USA) to extract total RNA. Using the SuperScript RIII First-Strand Kit (Invitrogen, USA), 20 µL of first-strand cDNA was produced in the end. Using matching specific primers, cDNA was amplified by PCR following reverse transcription. Sangon Biotechnology Co., Ltd (Shanghai, China) provided the primers. In a Bio-Rad S1000TM heat cycler (Bio-Rad, USA), 2× RealStar Fast SYBR qPCR Mix (A304-10, GenStar, China) was used for the q-PCR process. The 2^−ΔΔCt^ technique was used to examine the data. The sequences of all the primers used for qRT-PCR are listed as follows: *AURKB*: (Forward) 5′-CGCAGAGAGATCGAAATCCAG-3′ (Reverse) 5′-AGATCCTCCTCCGGTCATAAAA-3′; *GAPDH*: (Forward) 5′-GAAGGTGAAGGTCGGAG-3′ (Reverse) 5′-GAAGATGGTGATGGGATTTC-3′.

The reaction conditions were 95°C for 2 min; 95°C for 15 s, 60°C for 30 s, 40 cycles; melting curve 65–95°C, increment of 0.5, 5 s.


**Informed consent:** Informed consent has been obtained from all individuals included in this study.
**Ethical approval:** The research related to human use has been complied with all the relevant national regulations, institutional policies and in accordance with the tenets of the Helsinki Declaration, and has been approved by Ethics Committee of The First Affiliated Hospital of University of Science and Technology of China (Anhui Provincial Hospital).

### 2.2 Cell culture and transfection

The American Type Culture Collection provided the human trophoblast cell line HTR8/SVneo. At 37°C and 5% CO_2_, HTR-8/SVneo cells passaged three times were grown in DMEM media supplemented with 10% fetal bovine serum (Gibco, NY, USA) and 1% penicillin–streptomycin solution (Gibco, NY, USA). In the HG group, HTR-8/SVneo cells were exposed to 30 mM d-glucose for a duration of 72 h, while the control group received standard media treatment (5.5 mM d-glucose).

Using interferon reagent (Polyplus, New York, USA) and siRNA (Sequence designed by company, GenePharma, Shanghai, China) specific for AURKB, HTR8/SVneo cells were transfected in accordance with the manufacturer’s instructions. HTR8/SVneo cells should be cultured in a 24-well plate at a density of 1 × 10^5^ cells per well until 70–90% confluence is reached. Afterward, they were transfected with 50 nmol of AURKB and negative control using Lipofectamine 2000 reagent (Invitrogen, Carlsbad, CA) following the guidelines provided by the manufacturer. The effectiveness of silencing AURKB was verified by western blot analysis. The AURKB siRNA-1 sequence is 5′-GGUGAUUCACAGAGACAUA-3′.

### 2.3 Cell viability assay

We seeded 1 × 10^4^ cells in a 96-well plate. After 1 full day, 10 μL of Cell Counting Kit 8 (CCK8, Solarbio, Beijing, China) reagent was added to the culture. Subsequently, the cells were allowed to incubate for a complete hour. Subsequently, the absorbance at 450 nm was assessed using a microplate reader (DALB, Shanghai, China).

### 2.4 5-Ethynyl-2-deoxyuridine (EdU) incorporation assay

We utilized the Cell-Light EdU (Ribobio Co., Ltd) kit for cell staining. Briefly, cells (5,000 per well) were seeded into 96-well plates and given 12 h of EdU incubation. Following harvesting, the samples were washed in PBS, treated with paraformaldehyde, permeabilized in PBS containing 0.3% Triton X-100 for 10 min, and then rinsed. The cells were incubated with the Apollo staining reaction solution in darkness for 30 min. Subsequently, the nuclei were stained with DAPI for 5 min. The 2^−ΔΔCt^ technique was employed to analyze the relative differential gene expression.

### 2.5 Enzyme-linked immunosorbent assay (ELISA)

The ELISA method was used to determine the levels of interleukin 1β (IL-1β), interleukin 6 (IL-6), and tumor necrosis factor-α (TNF-α) in HTR8/SVneo cells. Briefly, HTR8/SVneo cells (1 × 10^5^) were seeded in six-well plates, and then cell lysates and supernatants were collected. Levels of IL-1β, IL-6, and TNF-α were assessed through ELISA kits from Jiangsu Meian Biotechnology Co., Ltd, following the prescribed protocols. Concentrations of cytokines were then calculated based on the standard curve derived from recombinant cytokines.

### 2.6 Western blot

HTR-8/SVneo cells’ proteins were separated using RIPA (Sigma, USA) buffer. The BCA assay (Thermo Fisher, A23227, USA) was used to measure the amount of protein. Following their separation using 10% SDS-PAGE, the extracted proteins were transferred to a polyvinylidene difluoride membrane (Millipore, 88,518, USA). Following the application of a 5% skim milk blocking solution, the primary antibodies were left to incubate in TBST buffer overnight at 4°C with gentle agitation. Subsequently, the samples underwent treatment with SuperSignalTM West Femto Chemiluminescent Substrate (Thermo Fisher, 34,096, USA) post incubation with the secondary antibody, specifically the horseradish peroxidase goat anti-rabbit IgG (1:3,000; Cell Signaling Technology, AB_2099233, USA). To record and analyze chemiluminescent signals ImageJ software was used. The following primary antibodies were used: AURKB (1:1,000, ab 218339; Abcam, USA), PI3K (1: 1,000, ab302958; Abcam, USA), p-PI3K (1:1,000, ab278545; Abcam, USA), Akt (1:1,000, ab8805; Abcam, USA), p-AKT (1:1,000, ab38449; Abcam, USA), and GAPDH (1:3,000, ab8245; Abcam, USA). All experiments were performed with at least three replicates.

### 2.7 Statistical analysis

The software program GraphPad Prism 8 (GraphPad Software, CA, USA) was utilized for statistical analyses. The Mann–Whitney *U* or Student’s *t* test was used to compare the means of the two groups in order to assess differences. One-way analysis of variance was used to compare the means of multiple groups. The standard deviation, or mean ± SD, is used to express all values. It was deemed statistically significant when *P* < 0.05.

## 3 Results

### 3.1 AURKB is highly expressed in HTR8/SVneo cells stimulated by HG

**Figure 1:**
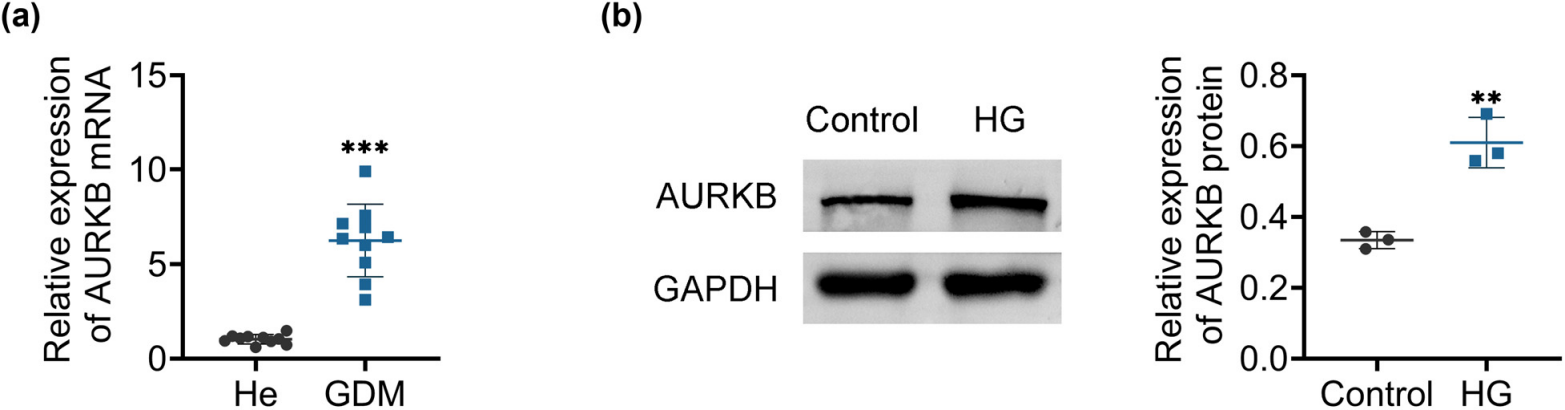
AURKB is highly expressed in HTR8/SVneo cells stimulated by high glucose. (a) AURKB levels were measured in the placental of healthy pregnant women and GDM pregnant women by RT-qPCR; *n* = 10. (b) Expression of AURKB in HTR8/SVneo cells; *n* = 3. Values are presented as mean ± SD. **p < 0.01, ***p < 0.001 versus He or control group.

In this work, we used RT-qPCR to show that, in comparison to healthy persons (He group), GDM patients (GDM group) had significantly higher maternal placental AURKB levels in late pregnancy (Figure 1a). We used western blotting to examine the expression levels of AURKB in HTR8/SVneo cells in both normal and HG settings. The results indicated that in HG conditions, the AURKB protein expression in HTR8/SVneo cells was significantly elevated compared to that in normal conditions (Figure 1b). The above findings indicate that AURKB may be involved in the onset and progression of GDM.

### 3.2 Knockdown of AURKB promotes growth in HG-stimulated HTR8/SVneo cells

**Figure 2:**
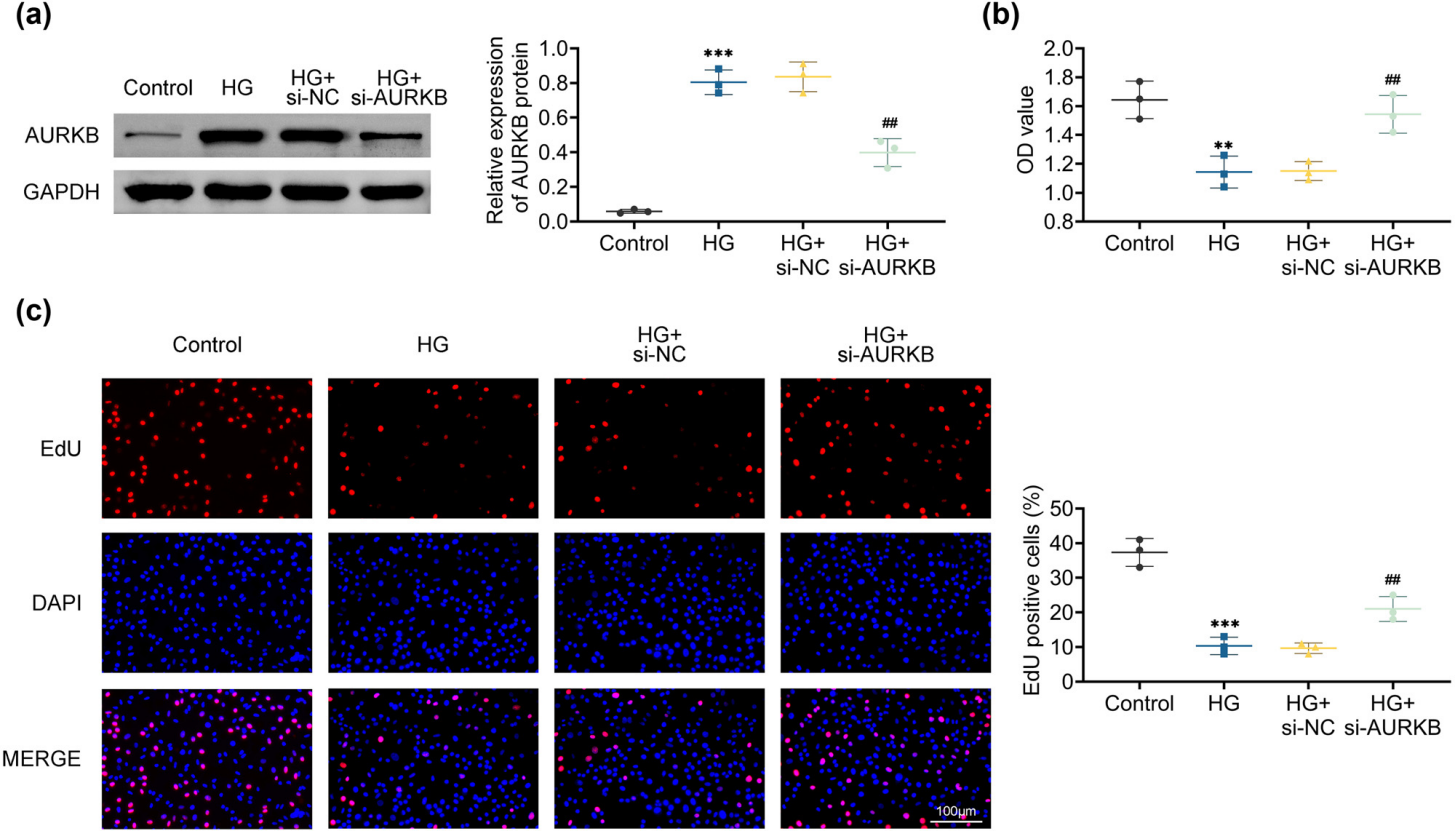
Knockdown of AURKB promotes growth in high glucose-stimulated HTR8/SVneo cells. (a) Expression of AURKB in HTR8/SVneo cells. (b) CCK8 detects the OD value of HTR8/SVneo cells. (c) EdU staining to detect the proliferation of HTR8/SVneo cells. Values are presented as mean ± SD. **p < 0.01, ***p < 0.001 versus HG + si-NC group. *n* = 3.

si-NC and si-AURKB were transfected, respectively, to investigate the impact of AURKB on HTR8/SVneo cells. Through western blotting analysis, it was verified that the knockdown of AURKB in HTR8/SVneo cells was effective, as evidenced by the reduced expression level of AURKB in the si-AURKB group, which was comparable to that of the control group and the si-NC group with no significant difference (Figure 2a). Next, by knocking down AURKB in HTR8/SVneo cells, we investigated the impact of AURKB on trophoblast cell proliferation. Knockdown of AURKB expression in HTR8/SVneo cells increases cell survival and proliferation, as demonstrated by CCK8 studies and EdU staining (Figure 2b and c). These findings suggest that AURKB plays a role in the impairment of trophoblast function.

### 3.3 Knockdown of AURKB inhibits inflammation in HG-stimulated HTR8/SVneo cells

**Figure 3:**
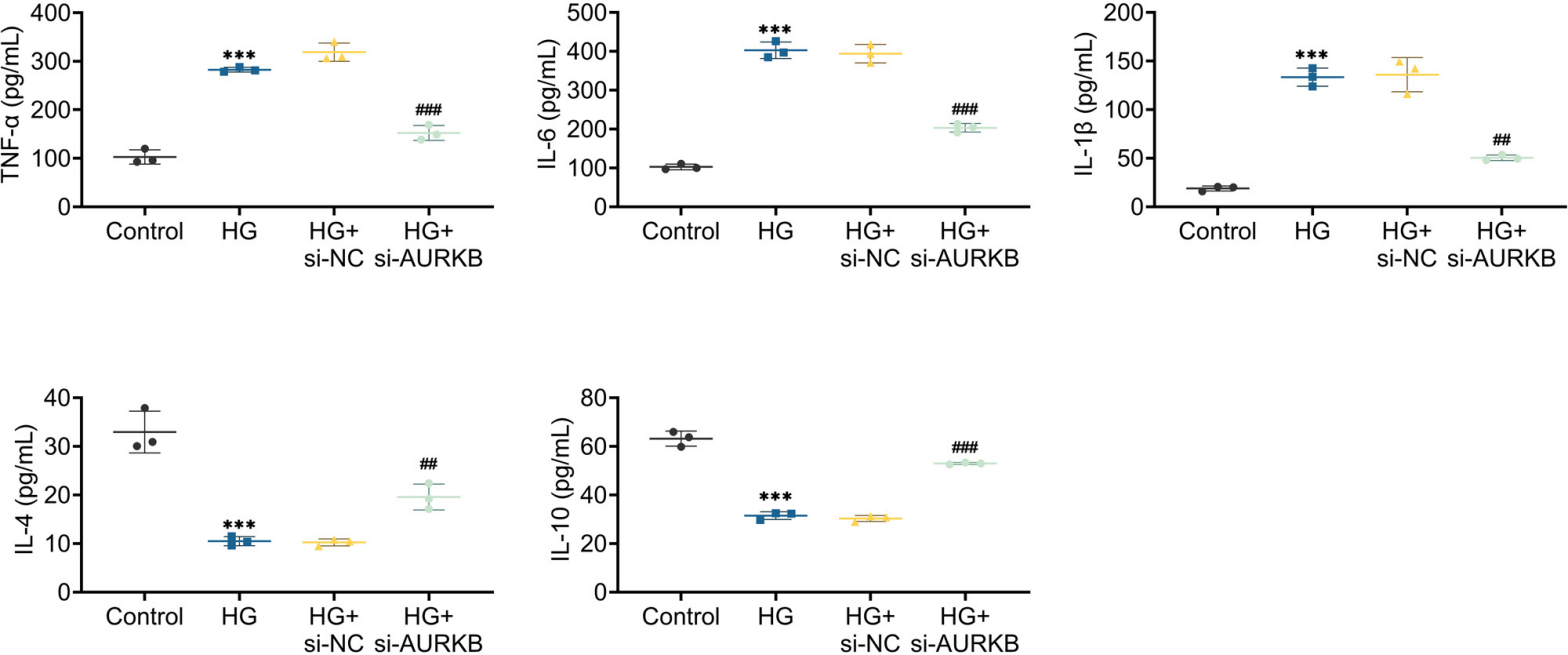
Knockdown of AURKB inhibits inflammation in high glucose-stimulated HTR8/SVneo cells. ELISA detects TNF-α, IL-6, IL-1β, IL-4, and IL-10 levels in HTR8/SVneo cells. Values are presented as mean ± SD. ***p < 0.001 versus HG + si-NC group. *n* = 3.

The impact of AURKB on trophoblast inflammation was further examined using AURKB knockdown in HTR8/SVneo cells. Analysis of ELISA data indicated that the suppression of AURKB could effectively impede the secretion of pro-inflammatory cytokines TNF-α, IL-6, and IL-1β under high-glucose conditions, while also inhibiting the synthesis of anti-inflammatory molecules IL-4 and IL-10 (Figure 3). The above findings suggest that AURKB is involved in trophoblast inflammation in HG environments.

### 3.4 Knockdown of AURKB inhibits the expression of PI3K/Akt signaling pathway

**Figure 4:**
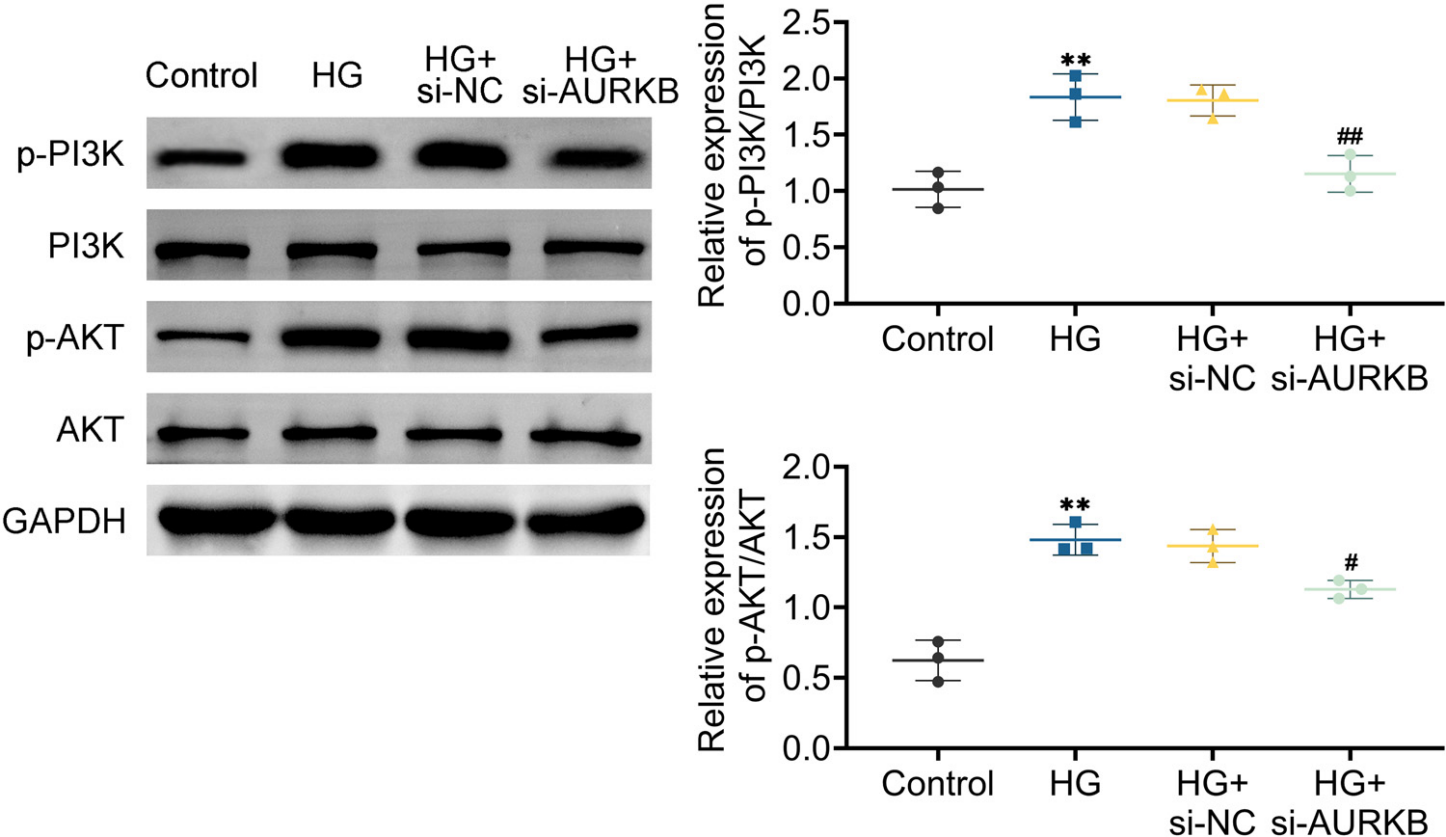
Knockdown of AURKB inhibits the expression of PI3K/AKT signaling pathway. WB detects the protein expression of PI3K, p-PI3K, AKT, and p-AKT in HTR8/SVneo cells. Values are presented as mean ± SD. **p < 0.01, ***p < 0.001 versus HG + si-NC group. *n* = 3.

We used western blot to compare the expression levels of several PI3K/AKT signaling components in order to comprehend the effect of AURKB on this signaling pathway. The results showed that in HG-induced HTR8/SVneo cells, knocking down AURKB inhibited the phosphorylation of PI3K and Akt proteins, and the PI3K/AKT pathway was inhibited (Figure 4). This indicates that AURKB might have a significant impact on HG-induced HTR8/SVneo cells, potentially by controlling the PI3K/Akt pathway.

## 4 Discussion

One of the common metabolic disorders linked to pregnancy is GDM, which leads to elevated levels of pro-inflammatory and anti-angiogenic factors. These substances impede trophoblast invasion, migration, and proliferation, thus establishing an unfavorable environment for the physical and psychological well-being of the fetus [17,18]. In addition, type 2 diabetes and metabolic syndrome are risk factors for later life in children (mothers with gestational diabetes). The reduced ability of the placenta to take up glucose has been increasingly associated with the emergence of metabolic issues in the children of mothers with GDM, although the specific mechanisms driving this phenomenon remain to be fully understood [3,19–21]. We then used serum from women with gestational diabetes and trophoblast cells grown in high-glucose conditions to perform RT-qPCR and western blotting experiments, which confirmed Zhao’s previous research by showing that HG levels increased the expression of AURKB [11].

AURKB is a crucial regulator of mitosis that is a member of the mitotic protein kinase family. It functions differently in healthy and pathological conditions [22,23]. While the detection of elevated levels of AURKB serves as a valuable prognostic and diagnostic indicator in clinical practice, as it has been observed in various forms of epithelial cancers [24]. The protein AURKB plays a significant role in controlling various signaling pathways that impact the growth, survival, invasion, mobility, and apoptosis of cancer cells [25]. Our research indicates that AURKB is highly active in GDM. Despite this, the specific way in which AURKB operates in GDM is still not well understood. In the early stages of pregnancy, the development of the embryo and placenta largely depends on trophoblasts, which are the key cells involved [26–29]. Recent laboratory experiments have revealed that reducing the activity of the AURKB gene can help protect trophoblast cells from damage and inflammation. Taken together, these results suggest that high levels of AURKB expression could play a role in causing trophoblast dysfunction in gestational diabetes.

We then looked at the potential mechanism by which AURKB knockdown benefited the GDM cell model in HTR-8/Svneo cells. The activation of the insulin signaling cascade is achieved through tyrosine phosphorylation of insulin receptor substrates, the autophosphorylation of receptor tyrosine residues, and insulin binding to the insulin receptor [30]. When the insulin receptor substrate-1’s tyrosine phosphorylation site binds to it, the signaling protein AKT is drawn toward the heterodimeric lipase PI3K. This lipase plays a crucial role in the metabolic processes involving proteins, lipids, and glucose [31]. The imbalance in the PI3K/AKT pathway, which is accountable for increasing cellular glucose uptake and managing cell growth, is strongly linked to GDM [32]. In this study, we found that knocking down can inhibit the phosphorylation expression of PI3K and Akt in trophoblast cells in a high-glucose environment.

## 5 Conclusion

According to our findings, trophoblast cells from GDM patients and those from high-glucose environments express more AURKB. In the pathogenesis of GDM, AURKB may play a role, and reducing its expression could potentially alleviate trophoblast cell damage and inflammation, at least partially by modulating the PI3K/AKT pathway. Further investigation is needed to elucidate the precise regulatory mechanism upstream of AURKB in GDM.
